# Posters

**DOI:** 10.1038/sj.bjc.6601935

**Published:** 2004-06-22


British Journal of Cancer (2004) 91(Suppl 1), S24 – S80
© 2004 Cancer Research UK All rights reserved 0007 – 0920/04 $30.00
www.bjcancer.com

### POSTERS

### P1

### PROSPECTIVE STUDY OF OUTCOMES IN SPORADIC VERSUS HEREDITARY BREAST CANCER

DM Eccles , PD Simmonds , SM Gerty
University of Southamtpon, Southampton, United Kingdom

This prospective cohort study has been recruiting women aged ≤ 40 years at breast cancer diagnosis since June 2001. MREC was amended to include women up to 50 years if they are known BRCA1 or BRCA2 gene carriers and diagnosed in the period since December 2001. Initial slow progress has been greatly facilitated since adoption into the National Cancer Research Network and a rapid increase in the rate of recruitment has occurred in the last year with over 50 hospitals now actively recruiting and the majority of cancer research networks active. Recruitment at the end of January 2004 stands at 432 with a steep rise in month on month recruitment in the last year with current recruitment at 50-60 patients per month. We have examined some of the available data on the characteristics of this unusual cohort. All patients have been diagnosed since December 2000. The average age at diagnosis is 35 (range 23-46). The heterozygote (HZ) risk (chance of carrying a high risk breast cancer susceptibility gene) for participants is derived from the reported family history and reveals that 11% have a HZ risk of ≥ 79% with a high likelihood of being BRCA1 or BRCA2 gene carriers (some are already known gene carriers). 65% have a HZ risk ≤ 21% and are most likely not to be due to major predisposition genes. The remaining 24% have an intermediate genetic risk amongst whom the proportion of BRCA1 and 2 gene carriers will be intermediate and in many cases disease will be due to polygenic influences. The vast majority of patients presented symptomatically. Tumour stage is similar in all risk groups but reported tumour grade for the high HZ risk patients had a higher proportion of high grade oestrogen receptor negative tumours than the other groups. Current studies planned using this cohort and executable during the accrual phase include a series of clinical comparisons between sporadic and hereditary groups and association studies to examine candidate gene polymorphisms for low penetrance susceptibility. The projected date for completion of the accrual of 2000 cases is June 2006, follow up will continue for a minimum of 5 years.

### P2

### TRENDS AND REPRODUCTIVE RISK FACTORS IN FEMALE BREAST CANCER INCIDENCE IN GREAT BRITAIN

P.S. Carroll
Pension and Population Research Institute, London, United Kingdom

National trends in incidence according to age of women are examined in relation to contemporary data on known risk factors for both the main jurisdictions in Great Britain for successive female birth cohorts born in the years since 1926. Forecast numbers of new malignant breast cancers for England & Wales and for Scotland for years up to 2026 are produced using a linear probability model fitted to national data on birth cohorts of the female population. The response variable is cumulated cohort breast cancer incidence within the ages 50 to 54. The two explanatory variables, used in the model as the best predictors among reproductive risk factors, were cumulated cohort rates of fertility and induced abortion. Allowance is also made for the additionally carcinogenic effect of nulliparous abortions. The analysis and modelling for Great Britain is compared with Sweden and the Czech Republic.

This modelling is also applicable to mortality trends as reported recently in Health Statistics Quarterly by ONS. [1] “In women, the inverse social class gradient in breast cancer increased…” The gradient across social class is explicable in terms of the same reproductive risk factors and a further increase in the inverse gradient can be anticipated.

The research updates research previous published [2] by the International Congress of Actuaries in Mexico in 2002, where the modelling focused on the incidence of breast cancer within the ages 45 to 49.

References:

1. Trends in social class differences in mortality by cause, 1986 to 2000. Chris White, Folkert van Galen, Yuan Huang Chow. Health Statistics Quarterly No 20. Winter 2003. ONS.
2. Pregnancy Related Risk Factors in Female Breast Cancer Incidence. Patrick Carroll. International Congress of Actuaries, Cancun, Mexico. Transactions (2002) 4:331-75

### P3

### USE OF MAGNETIC RESONANCE IMAGING TO PLAN BREAST CONSERVING SURGERY FOLLOWING NEOADJUVANT CHEMOTHERAPY FOR BREAST CANCER

M Bhattacharyya , S Vinnicombe , D Ryan , R Al-Mufti , R Carpenter, C Gallagher
St Bartholomew’s Hospital, London, United Kingdom

**Background**: Selection of patients (pts) for breast conserving surgery (BCS) following neoadjuvant chemotherapy (NAC) is dependant upon accurate assessment of the extent of residual disease within the breast Up to 60% patients fail primary surgery and require re-excision or mastectomy.

**Methods**: Pts seen over a 3-year period, with histologically confirmed invasive breast cancer on core biopsy, stage $cT_2 – T_3$, eligible for NAC and potentially suitable for BCS were included. Pre and post NAC mammography (MM), breast ultrasound (U/S), and MRI were independently reported and compared with clinical and pathological assessment.

**Results**: 32 pts median age 42 years were treated; 69% pre-menopausal; stage I 6%, II 62%, III 31%. Median tumour size pre-NAC was 4.75cm. All received 6 cycles of chemotherapy. Clinical response: CR 12.5%, PR 81.3% SD 6.2%. MM in this young population was uninformative in 62.5%. U/S and MRI correlation with pathological response was r= 0.65 and r=0.71 respectively. In 4 pts, MRI tumour size was > 1cm larger than pathological size due to associated DCIS in 3 and post treatment hyaline fibrosis in 1 patient. In 2 pts, MRI size was > 1cm smaller because of delayed surgery. MRI but not U/S was able to differentiate between residual invasive tumour and DCIS or fibrosis from the gadolinium enhancement curves.

Following NAC 25 (72%) were suitable for BCS (unsuitable- proximity to nipple 5, multifocal 1, skin tethered 1) of whom 20 proceeded (3 chose mastectomy, 2 radiotherapy only). 17 (85%) pts had successful initial surgery, 3 (15%)had positive margins requiring re-operation because of difficulty locating very small residual masses.

**Conclusion**: MRI aided the selection of pts for BCS after NAC due to the high correlation with pathological findings. 72% pts achieved BCS, 85% of them with single operation.

### P4

### DENDRITIC CELLS WITH IMMATURE PHENOTYPE AND DEFECTIVE FUNCTION IN SOLID TUMOURS FROM PATIENTS WITH BREAST CARCINOMA

S Islam [1], M Burke [1], SC Knight [2]
[1] Antigen Presentation Research Group, Imperial College London, London, United Kingdom, [2] Northwick Park Hospital, London, United Kingdom

**ABSTRACT WITHDRAWN**

### P5

**NAVELBINE ($N_V$) AND TRASTUZUMAB ($T_R$) IN COMBINATION FOR ADVANCED BREAST CANCER: A RETROSPECTIVE SINGLE INSTITUTION ANALYSIS**

T Benepal , G Attard , SRD Johnston , IE Smith
Royal Marsden Hospital, London, United Kingdom

$T_r$ in combination with Taxol is an accepted combination for use in advanced breast cancer. Non-randomised data also suggest that $N_v$ is active in combination with $T_r$. We performed a retrospective single institution analysis of 19 women with advanced breast cancer treated with the combination over a 20 month period from May 2002 to December 2003. Their median age was 55 yrs (range: 25-74). All the patients treated had evidence of disease progression prior to therapy. All tumours were confirmed, prior to treatment, to be Her2 3+ by immunohistochemistry. Thirty seven percent (7/19) were ER negative. Sixteen percent (3/19) were grade 1 ER positive, 26% (5/19) grade 2 ER positive, and 21% (4/19) grade 3 ER positive on staining. A median of 6 cycles of chemotherapy were given (range 1-8). All patients were scheduled to receive $N_v$ at 25 mg $m^{-2}$ days 1 and 8 of a 21 day cycle. $T_r$ was given weekly at 2 mg $kg^{-1}$ in 15 patients and three weekly at 6 mg $kg^{-1}$ in the other 4. One patient received the combination 1st line; seven 2nd line, nine 3rd line and one 4th line. The regimen was well tolerated: neutropaenia was the only grade 3 or 4 toxicity and occurred in 5 patients. Lethargy was the commonest non-haematological toxicity. Forty seven percent (9/19) of patients responded to treatment, including 6 who were ER negative. A further 21% (4/19) had stable disease, and 32% (6/19) progressed on treatment. Of the 10 patients with stable or progressive disease only 1 was ER negative. We conclude that this combination is well tolerated and this small series suggests that it may be more effective in patients with ER negative tumours. This issue should be further assessed in other series.

### P6

**AGE INFLUENCES THE BIOLOGY OF PRIMARY BREAST CANCER**

JR Buckley , GA Thomas , K Malinovszky , C Jones , RCF Leonard
Cancer Institute Swansea University, Swansea, United Kingdom

Factors that have been found to relate to the prognosis of breast cancer include tumour size, node status and oestrogen receptor (ER) status. With changing demographics and a consequent interest in managing disease in the aging cancer population, there is a need to ascertain biological differences between cancers of the young and old. These findings may have implications for targeting treatment. The dividing line between pre- and post-menopausal cases is a significant one when deciding about treatment policy. Eppenberger-Castori et al (2002) studied a large group of cases comparing different age-groups in terms of biological markers and found that there was an association between some biological markers and age.

We determined to do similarly, but selected extremes of the age spectrum by studying local cases of the disease. We compared a young pre-menopausal group (aged under 43) with two older post-menopausal groups (aged 70 to 72 and over 84 respectively, subsequently, because of their similar findings, combining them to form one elderly post-menopausal group).

We found statistically significant positive correlations between advancing age and ER status ($\chi^2 = 13.8579$, $P < 0.001$) and node positivity ($\chi^2 = 5.6559$, $0.01 < P < 0.025$). There were positive trends between advancing age and increasing tumour size and Nottingham Prognostic Index (NPI). Statistically significant negative correlations were found between advancing age and grade ($\chi^2 = 15.0567$, $P < 0.001$).

In conclusion, despite an increased ER positivity, older patients have a poorer prognosis for cause-specific survival based on these features, which is reflected by NPI score. Therefore, there are marked differences in tumour biology between young and old.

### P7

**LOW 25HYDROXYVITAMIN $D_3$ LEVELS AND BREAST CANCER RISK IN A UK CAUCASIAN POPULATION**

LC Lowe [1], M Guy [1], K Townsend [2], MJ Campbell [2], M Hewison [2], JL Mansi [1], KW Colston [1]
[1] St. George's Hospital Medical School, London, United Kingdom,
[2] The University of Birmingham, Birmingham, United Kingdom

The active form of vitamin $D_3$, 1,25dihydroxyvitamin$D_3$ (1,25-$D_3$) is known to have anti-carcinogenic properties in various cancer cell types, including breast. 1,25-$D_3$ is produced from 25hydroxyvitamin$D_3$ (25-$D_3$) by the enzyme 1 hydroxylase (1 OHase, encoded by the Cyp27b gene). While 1 OHase was thought to be exclusively a renal enzyme, recent evidence has shown it is active in other tissues, suggesting local 1,25-$D_3$ production at sites other than the kidney. Our aim was to determine if breast tissue expresses 1 OHase and whether breast cancer patients have lower levels of circulating 25-$D_3$ than controls. Paired normal and tumour breast tissue samples from 41 Caucasian patients (taken with full ethical approval) were analysed for Cyp27b mRNA expression using real time RT-PCR. Normal breast exhibited strong expression of Cyp27b and this expression was increased in oestrogen receptor (ER) positive tumours (mean = 23 fold increase, P<0.0001, n=29) and ER negative tumours (mean = 57 fold increase, P<0.0001, n=12). This indicates that 1,25-$D_3$ may be generated locally in the breast and that circulating 25-$D_3$ levels may influence its production. Circulating 25-$D_3$ levels were measured by ELISA in 179 Caucasian breast cancer patients recruited with full ethical approval and 179 Caucasian control volunteers, matched for time of year, age and menopausal status. Breast cancer patients had significantly lower 25-$D_3$ levels than controls (80.13nM +/- 43.44 vs. 97.6nM +/- 40.87, p=0.0001). Furthermore, when all values were separated into quartiles, it was found that women in the lowest quartile had an odds ratio (OR) of 5.19 (95% confidence interval 2.23-12.08, p=0.001) for breast cancer risk compared to women in the highest quartile. In conclusion we hypothesise that low circulating levels of 25-$D_3$ diminishes the ability of the breast to locally produce 1,25-$D_3$ and so may contribute to breast cancer development. 25-$D_3$ measurements may serve as a marker for women at higher risk of breast cancer.

### P8

**THE 5A/6A POLYMORPHISM OF THE STROMELYSIN I GENE PROMOTOR AND BREAST CANCER**

P Krippl , U Langsenlehner , T Eder , H Samonigg
Division of Oncology, Medical University Graz, Graz, Austria

Purpose: The matrix metalloproteinase 3 (MMP3), also known as stromelysin-I, is a key-player for carcinogenesis and tumor growth. A 5A/6A promoter polymorphism is associated with differences in MMP3 activity and has been linked to cancer susceptibility in some studies. In the present study we evaluated the role of this polymorphism for breast cancer risk.

Patients and Methods: A case-control study was performed including 500 patients with histologically confirmed breast cancer and 500 female, age-matched, healthy control subjects from population-based screening studies. The MMP3 5A/6A polymorphism was determined by a 5'-nuclease (TaqMan) assay.

Results: Prevalences of 5A/5A, 5A/6A and 6A/6A genotypes were similar among patients (20.6%, 51.8%, 27.6%) and controls (23.3%, 47.3%, 29.4%, p = 0.34). The odds ratio of carriers of a MMP3 5A allele for breast cancer was 1.09 (95% confidence interval 0.83 - 1.44). Patients with the 5A/5A genotype had a higher proportion of lymph-node metastases than those with a 5A/6A or 6A/6A genotype (P = 0.010).

Conclusion: The MMP3 5A/6A promoter polymorphism does not appear to influence breast cancer susceptibility, but may be linked to a higher risk for metastasizing among breast cancer patients.

© 2004 Cancer Research UK

### P9

### THE SULT1A1 213H VARIANT IS ASSOCIATED WITH THE PRESENCE OF LYMPH NODE METASTASES IN BREAST CANCER PATIENTS

U Langsenlehner , P Krippl , T Eder , H Samonigg
Division of Oncology; Medical University Graz, Graz, Austria

Background: Sulfotransferase 1A1 (SULT1A1) is involved in the bioactivation and detoxification of a variety of xenobiotic compounds, especially in the inactivation of estrogens. A common arginine (R) to histidine (H) variation at amino acid position 213 influences SULT1A1 activity and has been suggested as a risk factor for a variety of cancers.
Patients and methods: To investigate the role of this polymorphism for breast cancer risk, SULT1A1 genotype was determined in 500 women with clinically verified breast cancer and 500 female age-matched healthy control subjects. Genotyping was done by a 5'-nuclease assay (TaqMan™). Primer and probe sets were designed and manufactured using Applied Biosystems 'Assay-by-Design' custom service. P-values were calculated by $\chi^2$ test using SPSS 10.0. All tests were two-sided, threshold for significance was $P < 0.05$.
Results: Frequencies of heterozygous (controls: 42.5%; patients: 50.2%) or homozygous (controls: 12.6%; patients: 9.4%) carriers of the 213H variant were not significantly different between groups. The odds ratio of the SULT1A1 R213H variant was 1.20 (95% confidence interval 0.94 – 1.55) in a dominant model (RH+HH versus RR) and 0.72 (95% confidence interval 0.48 – 1.08) in a recessive model (HH versus RH+RR), respectively.The SULT1A1 genotype was furthermore not associated with tumor size, histological grading, estrogen or progesterone receptor status and age at diagnosis. The SULT1A1 213H variant was associated with the presence of lymph node metastases ($p = 0.002$).
Conclusion: We conclude that the SULT1A1 R213H polymorphism is not a general risk factor for breast cancer, but may be involved in lymph node metastazing in breast cancer patients. Further prospective studies should be performed to analyze this potential role of the SULTA1 polymorphism for lymph node metastasis development.

### P10

### THE 677C>T POLYMORPHISM OF METHYLENETETRAHYDRO FOLATE REDUCTASE GENE AND SUSCEPTIBILITY TO BREAST CANCER

U Langsenlehner , P Krippl , H Samonigg
Division of Oncology, Medical University Graz, Graz, Austria

Introduction: Methylenetetrahydrofolate reductase (MTHFR) is involved in folate metabolism and plays a role in DNA biosynthesis, methylation, and repair in actively dividing cells. A common 677C>T polymorphism in the gene for MTHFR, leading to a thermolabile enzyme with decreased activity, has been associated with reduced plasma folate levels and elevated homocysteine levels and could be a risk factor for breast cancer.
Patients and Methods: In the present case-control study, MTHFR genotype was determined in 500 women with clinically verified breast cancer and 500 female age-matched healthy control subjects. A fragment containing the polymorphic site was amplified by polymerase chain reaction. Statistic analysis was done using SPSS 11.0 for Windows. Numeric values were analyzed by Student's t-test, proportions of groups were compared by $\chi^2$ test.
Results: The homozygous TT genotype was found in 13.0% patients and 13.1% controls (p = n.s.). The odds ratio of TT homozygotes for breast cancer was 0.99 (95% confidence interval 0.68 – 1.43). The MTHFR genotype was furthermore not associated with tumor size, histological grading, estrogen or progesterone receptor status and age at diagnosis. In a subgroup of 116 premenopausal patients, no increased frequency of the homozygous 677T genotype was found (13.8%).
Conclusion: Therefore, we conclude that the MTHFR 677C>T polymorphism is not associated with individual susceptibility to breast cancer.

### P11

### THE EFFECTS OF A NOVEL CDK INHIBITOR ON TAMOXIFEN SENSITIVE AND RESISTANT BREAST CANCER CELL LINES

N Johnson , N Curtin
Northern Institute for Cancer Research, University of Newcastle Upon Tyne, Newcastle, United Kingdom

Antiestrogens such as tamoxifen have been the standard treatment for estrogen receptor (ER) positive breast cancers. Tamoxifen antagonises estrogen – ER interactions resulting in down regulation of ER-mediated gene transcription which results in p27 accumulation. p27 inhibits CDK2/cyclinE/A complexes involved in the G1/S transition. Elevation of p27 levels by tamoxifen causes cell cycle arrest and inhibition of breast cancer cell growth. However, less than half of breast cancers respond to tamoxifen therapy and antiestrogen resistance usually develops. Direct inhibition of CDK2 may inhibit the growth of hormone refractory as well as hormone responsive breast cancers. The novel CDK inhibitor NU2058 inhibits the activity of CDK2 ($IC_{50}$ 16.6 ± 2 μM) and CDK1 ($IC_{50}$ 26 ± 3.5 μM) and was investigated in breast cancers with different sensitivities to tamoxifen.
The ER status of MCF7 and MCF7/LCC9 cells (a tamoxifen resistant MCF7 derived cell line) were measured (western blot) to give an indication of the importance of ER levels in predicting tamoxifen sensitivity. ER expression was more than twice as high in MCF7 than MCF7/LCC9 cells. In 6 day growth inhibition experiments (SRB) tamoxifen was 4 times as potent in MCF7 than in MCF7/LCC9 cells but NU2058 was equally effective in both cell lines (see table).

| Cell line | ER (fmoles/mg cellular protein) | Tamoxifen $GI_{50}$ (μM) | NU2058 $GI_{50}$ (μM) |
|---|---|---|---|
| MCF7 | 124 ± 74 | 2 ± 0.4 | 27 ± 3 |
| MCF7/LCC9 | 55 ± 32 | 8 ± 1.2 | 34 ± 10 |

In conclusion, tamoxifen effects on cellular proliferation are more pronounced in tamoxifen sensitive MCF7 cells than in tamoxifen resistant MCF7/LCC9 cells. In contrast, the effects of NU2058 were similar in both cell lines.

### P12

### IS HIC1 (HYPEMETHYLATED IN CANCER 1) A THERAPEUTIC TARGET IN BREAST CANCER?

H McDowell [1], G Nicoll [1], G Thomson [2], NM Kernohan [2], D Martin [2], AM Thompson [1]
[1] Department of Surgery and Molecular Oncology, University of Dundee, Dundee, United Kingdom, [2] MRC/CRUK Tissue Bank, University of Dundee, Dundee, United Kingdom

A transcriptional repressor gene, HIC1 (hypermethylated in cancer), on chromosome 17p may be of clinical importance with therapeutic potential in sporadic breast cancer. Preliminary data from our laboratory demonstrated failure of HIC1 expression in 86% of fifty breast cancers but conversely, HIC1 expression appeared to be associated with good prognostic features. The aim of this study was to confirm these findings on a second series of breast cancers and to compare HIC1 expression to p53 function since p53 may transcriptionally activate HIC1.
Based on 250 breast cancer samples, HIC expression was successfully determined in 153 cancers using polymerase chain reaction (PCR) based techniques and identified at normal levels or overexpressed in the cancer from 14/153 (9%) patients. In these 14 patients, the genomic HIC1 DNA failed to show mutation using Wave but p53 mutation was identified with the yeast functional assay and confirmed by sequencing in 4 tumours. The same series of 250 cancers was examined for p53 protein expression using immunohistochemistry with antibodies DO1, DO7, Ser15 and FP3.
Comparison of HIC1 gene expression as a continuous or dichotomous variable with clinical and pathological parameters demonstrated no statistically significant associations. There was also no association between HIC1 expression and p53 immunohistochemistry.
This study confirmed suppression of HIC1 in the majority (86%) of breast cancers. However, where HIC1 expression remained, neither HIC1 nor p53 were commonly mutated and we thus conclude that HIC1 may be a bystander in breast cancer rather than a suitable target for novel therapeutic intervention.

© 2004 Cancer Research UK

### P13

### BREAST CANCER AND HORMONE THERAPY IN POSTMENOPAUSAL WOMEN

LE Turner [1], NJ Bundred [2]
[1] The University of Manchester, Manchester, United Kingdom,
[2] Academic Surgery, South Manchester University Hospital, Manchester, United Kingdom

**Introduction:** Menopausal symptoms such as hot flushes, night sweats and osteoporosis lead to considerable morbidity in the Western World. Women with breast cancer often develop an early menopause due to treatment effects such as chemotherapy. Osteoporosis causes fractures in around 3-5% of breast cancer patients on adjuvant endocrine therapy (particularly after aromatase inhibitor therapy). Hormone replacement therapy (HRT) is effective in relieving menopausal symptoms but its use is generally discouraged in women with breast cancer.
**Aims:** To determine the incidence of menopausal symptoms and to identify what factors predicted HRT use in women with breast cancer compared to a control group. The frequency of osteoporosis or osteopaenia was determined in a sub-group of 70 women with early breast cancer who had recent Lunar Hologic Dual Energy X-ray Absorptiometry (DEXA).
**Results:** Incidence and severity of menopausal symptoms were greater in 146 women with breast cancer, particularly those on adjuvant therapy or after chemotherapy compared to 133 controls ($p<0.05$). Psychosocial problems such as depression, state anxiety and loss of sexual interest were significantly dependent of group ($p<0.05$). Factors associated with HRT use were the need for effective treatment of symptoms (positive correlation, p=0.01) and women with breast cancer (negative association, p=0.03). 4% of the sub-group were osteoporotic and 33% were osteopaenic. Of women who were 60 years or older, 38% were osteopaenic, of those who were smokers (previous or current), 50% were osteopaenic compared to 25% of non-smokers ($p>0.05$).
**Conclusion:** For women with breast cancer the menopause has a huge impact on quality of life. Women in whom adjuvant aromatase inhibitor is proposed require baseline bone mineral density scanning prior to its commencement. Screening menopausal symptoms is imperative to address the needs of women with breast cancer.

### P14

### CENTRAL NERVOUS SYSTEM METASTASES IN WOMEN WITH METASTATIC BREAST CANCER

M Hawkins, S Lalondrelle, AJ Neal
United Kingdom, St.Luke's Cancer Centre, Guildford

**Introduction** Trastuzumab (T) is the drug of choice in women with metastatic breast cancer overexpressing HER-2. T does not cross the blood-brain barrier.
**Methods** This is a retrospective study looking at a cohort of patients with metastatic breast cancer (MBC) in St. Luke's Cancer Centre Guildford over the period March1999-March 2003. 68 patients with HER-2 3+ MBC who have received T alone or in combination have been identified, but only 60 had adequate details. None of the patients had central nervous system disease (CNS) prior to starting T. The median follow-up of the cohort was 24 weeks from commencing T.
**Results** 16 patients (26.6%) developed brain metastases (BM) while on T. Median time follow up on T was 44 weeks in patients who developed BM versus 20 weeks in patients who did not. 13 out of 16 patients who developed BM had partial response or stable systemic disease when BM was diagnosed. Following the diagnosis of BM, all patients received whole brain radiotherapy. Median survival following diagnosis of BM was 6.5 months. 11 out of 16 patients died of progressive brain disease following the diagnosis of BM. The initial disease free interval from diagnosis of cancer was longer in patients who developed BM: 32.5 months versus 21 months. There were 16 deaths in the non-BM patients due to liver failure and the median duration of treatment in this group was only 14 weeks.
**Conclusion** T is effective in treatment of HER2 3+ patients. There is a group of patients who respond well and despite having stable visceral and bone disease, develop BM ultimately causing mortality. Interestingly another group of patients has been identified who has different characteristics: shorter disease free interval from the diagnosis of breast cancer, and in which once metastatic disease developed response to T is brief and death occurs due to liver or multiorgan failure.

### P15

### TIME AND CHEMOTHERAPY TREATMENTS TRENDS IN THE ELDERLY (AGE>70) WITH LUNG CANCER IN A SINGLE UK CANCER CENTRE

Yau Thomas, Asley Sue, Norton Alison, Athena Matakiou, Popat Sanjoy, David Watkins, Tim Eisen, Simth IE, O'Brien Mary
Royal Marsden Hospital, London, United Kingdom

**Background:** There are few data on the outcome in elderly patients(>70) with lung cancer over the past 20 years. Equally, there is little know about long term outcomes when treated with chemotherapy. This study focuses on elderly lung cancer patients(age>70) in a single UK cancer centre treated over the past 20 years.
**Methods:** We retrospectively reviewed 90 patients who received treatment for lung cancer(NSCLC,SCLC and mesothelioma)at the Royal Marsden Hospital(RMH) between 1982 and 2003. Patient characteristics, type of tumours, stage, performance status(PS), year of presentation, treatment and cause of death were analyzed. Survival analysis was done by Kaplan Meier method.Comparion of all cause survival was done between patients presenting in 1982-1994 and 1995-2003 by the log rank test. Comparison of survival between different treatment regimes used the proportional hazards model.
**Results:** There has been an improvement in all cause survival(adjusted for sex,stage and PS) in all age groups. Between 1982 and 2003, there were 960 elderly patients:519 aged 70-74, 328 aged 75-79 and 113 aged >80. Of these, 55% of patients had NSCLC, 38 % SCLC and 7% patients had mesothelioma. Over 85% patients were had performance status 0-2. In the early cohort, there were 270 patients and 690 in the most recent cohort. The later group of has better survival in all age groups and in particular in patients>80($p<0.001$). The finding is valid in patients with NSCLC(p=0.008) and SCLC(P=0.0006). As treatment, 41% patients received a cisplatin combination, 18% a carboplatin combination, 4% a non-platinum combination, 17% single agent and 30 % no chemotherapy.Carboplatin combinations were significantly better than others after adjusting for PS , stage and sex(p=0.007).Cisplatin combinations were found to do marginally better than non-platinum combinations(p=0.06).
**Conclusion:** This analysis demonstrated that there has been a significant improvement in survival for elderly patients with lung cancer in past 20 years after adjustment for stage and PS.Carboplatin combinations seems to have a greater impact on survival than other combinations or single agent treatment.

### P16

### A RANDOMISED TRIAL OF EARLY VERSUS DELAYED CHEMOTHERAPY IN SYMPTOMATICALLY STABLE PATIENTS WITH MALIGNANT MESOTHELIOMA

D Watkins [1], M O'Brien [1], S Ashley [1], A Norton [1], C Ryan [2], A Saini [2], M James [1], K Priest [1], T Eisen [1], I Smith [1]
[1] United Kingdom, Royal Marsden Hospital, Sutton, [2] United Kingdom, Kent Oncology Centre, Maidstone

**Background:** Prior phase II trials have demonstrated the therapeutic activity of cytotoxic chemotherapy in mesothelioma. Currently there is little randomised data assessing the role of chemotherapy vs best supportive care (BSC). In the management of patients (pts) with stable symptoms, a policy of observation may be adopted over initial use of chemotherapy. In this prospective randomised trial we assess the use of early vs delayed cytotoxic therapy. The study opened in 1998, and closed in view of a competing national study (MSO1) in 2003.
**Methods:** Eligible pts were; Performance status (PS) 2, life expectancy >3 months and had stable symptoms for at least 4 weeks prior to randomisation. Pts were randomised to receive immediate chemotherapy or initial BSC with the addition of chemotherapy at time of symptomatic progression. All pts received the same platinum based chemotherapy regimen; MVP / MVCarbo (Mitomycin C 8mg/m$^2$ cycles 1,2,4 & 6, Vinblastine 6mg/m$^2$, max 10mg, and Cisplatin 50mg/m$^2$ (or Carboplatin AUC 5) every 3 weeks for up to 4-6 cycles.
**Results:** A total of 41 pts were recruited. Data are available on 39 pts, 19 of which were randomised to the early treatment (ET) group and 20 to the delayed treatment (DT) group. The median age for the ET and DT groups were 58 years (range 50-78) and 67 years (range 48-75) respectively (p=0.07). PS was 0 or 1 in 12 of the ET group and 14 in the DT group. All 19 pts in the early group received chemotherapy vs 15 pts in the delayed group. A total of 65 cycles of chemotherapy were delivered in the ET group and 40 cycles in the DT group. Median time to symptomatic progression was 25 weeks in the ET group compared to 11 weeks for the DT group (p=0.04). Median survival was 12 month for the early group compared to 10 months for the late group (p=0.01).
**Conclusion:** In this patient group, presenting with initial stable symptoms, the early use of chemotherapy provided an extended period of symptom control, and in this small trial, a survival advantage.

© 2004 Cancer Research UK

### P17

### FAMILIAL LUNG CANCER RISKS: RESULTS FROM THE GELCAPS STUDY

A Matakidou [1], T Eisen [1], M O'Brien [2], H Bridle [1], R Mutch [1], R S Houlston [1]
[1] United Kingdom, Sections of Cancer Genetics and medicine, Institute of Cancer Research, Sutton, [2] United Kingdom, Lung Unit, Royal Marsden Hospital, Sutton

Lung cancer is the leading cause of cancer death in the UK. Cigarette smoking has long been established as the predominant risk factor for lung cancer with an estimated 90% of cases being attributed to tobacco exposure. Lung cancer was first noted to aggregate in families in the 1960s. Familial aggregation of lung cancer in the UK was examined by means of a nationwide, case-control study (GELCAPS) conducted between 1999 and 2004. We compared lung cancer prevalence in first-degree relatives of 1004 female patients and 954 female controls. Overall, lung cancer in parents or siblings was associated with a 1.51-fold (95% confidence interval (CI): 1.12-2.04) increase in lung cancer risk. For the younger participants (below age 61) the risk was 1.95 (95% CI: 1.17-3.24). Analysis by histological subtype identified the highest risk in relatives of patients with a diagnosis of squamous cell lung cancer (OR=1.83, 95% CI: 1.22-2.73) and the lowest in those with small-cell lung cancer (OR=1.24, 95% CI: 0.78-1.97). Further analysis will be presented in the meeting. Results confirm previous findings and support the etiologic role of a genetic predisposition to lung cancer.

### P18

### CELL CYCLE REGULATORY PROTEINS AS THERAPEUTIC TARGETS: A VALIDATED METHOD FOR PHARMACODYNAMIC EVALUATION OF CLINICAL TRIALS

P Bradbury [1], C Norbury [2], A Olsen [3], M Slade [4], E Cattell [1], D Talbot [1]
[1] Cancer Research-UK Medical Oncology Unit, Oxford, United Kingdom, [2] Sir William Dunn School of Pathology University of Oxford, Oxford, United Kingdom, [3] Weatherall Institute of Molecular Medicine, Oxford, United Kingdom, [4] OSLER Chest Unit The Churchill Hospital, Oxford, United Kingdom

**Background:** Agents that target cell cycle regulatory proteins are increasingly being developed to treat cancer. A necessary part of the clinical development of such agents is proving the mechanism of action at the cellular level. We report a simple method of validating the mechanism of action of cell cycle specific drugs using FACS analysis of endobronchial tumour samples.
**Methods:** We enrolled informed consenting patients with non-small cell lung cancer (NSCLC) participating in clinical trials of agents that induce apoptosis through inhibition of cell cycle proteins. Cell cycle analysis was performed on bronchial washings (BW), bronchial brushings, (BB) and biopsy (BX) aspirates of metastases before and after treatment. BW, BB and tumour aspirates were fixed in 70% ethanol, stained with propidium iodide and analysed by flow cytometry. BX were desegregated in PBS/trypsin/collagenase before ethanol fixation. Red fluorescence (DNA) data were collected on samples of 10,000 cells. DNA was assessed as apoptotic (<G1 content), diploid (G1 content) or cycling/aneuploid (>G1). BW and BB from the contralatereal unaffected lung were used as controls.
**Results:** 23 patients with NSCLC have been studied. BB, BW and BX but not aspirates generated sufficient material for analysis. Tumour cells exhibited a large variability in aneuploidy compared with normal bronchial mucosa. Similarly, tumour cells had a greater <G1(apoptotic) fraction than controls. Treatment caused a large decrease in the >G1 population of tumour cells and corresponding increase in <G1 fraction. For BX a low proportion of diploid cells and high levels of presumptively apoptotic cells were seen prior to treatment: This fraction increased markedly after treatment. These analyses were more sensitive than radiological response to treatment.
**Conclusion:** FACS analysis of tissue obtained from BB provides a minimally invasive but robust, quantitative method for confirming the mechanism of action of novel agents at the cellular level.

### P19

### A PROSPECTIVE STUDY OF THE INCIDENCE OF SUB-CLINICAL LAMBERT-EATON MYASTHENIC SYNDROME (LEMS) IN PATIENTS WITH SMALL CELL LUNG CANCER (SCLC)

P Bradbury [1], B Lang [2], A Vincent [2], C Han [1], D Talbot [1]
[1] Cancer Research-UK Medical Oncology Unit, Oxford, United Kingdom, [2] Neurosciences Group Weatherall Institute of Molecular Medicine, Oxford, United Kingdom

**Background:** LEMS is one of the most common paraneoplastic disorders associated with SCLC. Autoantibodies targeted to the P/Q type voltage gated calcium channel (VGCC) are believed to be a cause of LEMS and form the basis of a diagnostic serological test. It is not known how many patients (pts) presenting with SCLC have positive anti-VGCC antibodies (Ab) and subclinical LEMS who may then be susceptible to develop clinical LEMS. We used a sensitive serological test to determine, in a prospective manner, the incidence of subclinical LEMS in SCLC.
**Methods:** Consenting Pts with a pathological diagnosis of SCLC completed a symptom questionnaire, had clinical examination for signs of LEMS, and serum for P/Q anti-VGCC Ab was taken. VGCC Abs were measured by immunoprecipitation of
[$I^{125}$ ]-labelled conotoxin MVIIC bound to VGCCs extracted from healthy cerebellum. Antibody titres were considered positive if >50pM (Mean +3SD for healthy controls n=30). Multivariate regression was used to determine the relationship between clinical symptoms and anti-VGCC titre, Cox analysis to evaluate survival.
**Results:** Of the 64 pts (46 male 18 female) tested, 5 (8%) pts had elevation of the anti-VGCC antibodies with levels ranging from 60-1553pM. Only one had confirmed LEMS on clinical grounds and confirmed by electrophysiological tests. The other 4 patients did not have clinical evidence of LEMS. There was no association between anti-VGCC titre and survival (p=0.08, hazards ratio 0.999)
**Conclusions:** This prospective study indicated that increased titre of anti-VGCC antibodies is common in SCLC. The presence of antibody positivity may help to identify patients who are at risk of developing the debilitating symptoms of LEMS.

### P20

### CYCLOOXYGENASE-2 PREDICTS SURVIVAL IN MALIGNANT PLEURAL MESOTHELIOMA

SL O'Kane , A Campbell , MJ Lind , L Cawkwell
PGMI, University of Hull, Hull, United Kingdom

Mesothelioma is a relatively rare, aggressive disease affecting the mesothelium. In the past, many cases of mesothelioma may have been misdiagnosed, mainly as lung cancer. Nonsteroidal anti-inflammatory drugs (NSAIDs) are known to decrease the incidence of cancer. One type of NSAID is the cyclo-oxygenase (Cox) inhibitors. The Cox enzymes are involved in the production of prostaglandins from arachidonic acid. There are three known isoforms of the Cox enzyme: Cox-1, Cox-2, and the recently identified Cox-3. Cox 1 which is inhibited by aspirin for example, is found in normal tissue and at the site of inflammation. Cox-2 is inducible and found in neoplastic tissue. Rofecoxib is an example of a Cox-2 inhibitor. Cox-2 has been described as being overexpressed in a small study of fresh mesothelioma samples. We aimed to assess Cox-2 on a larger series of archival formalin fixed specimens.
Formalin-fixed paraffin embedded resection/biopsy blocks were obtained from forty five malignant pleural mesothelioma cases (27 epithelial subtype, 8 sarcomatoid subtype and 10 biphasic subtype). Expression of Cox-2 was detected by immunohistochemical analysis, carried out on 4μm sections using a monoclonal anti-Cox-2 antibody.
The sections stained with varying intensity and staining was located in the cytoplasm of the tumour cells. Positive staining of the reactive spindle cells in the sections acted as a positive internal control. Overall 70% of tumour sections were positive. All sections were independently scored by two investigators.
Survival was calculated from date of diagnosis to date of death and analysis was performed with SPSS version 11.0 using a Kaplan Meier curve. The results indicate that overexpression of Cox-2 in malignant pleural mesothelioma is significantly related to prognosis (p = 0.0171) and therefore is worthy of further follow up by increasing the number of cases.

© 2004 Cancer Research UK

### P21

### GEFITINIB (IRESSA) IN ADVANCED NON SMALL CELL LUNG CANCER (NSCLC): RESPONSE, SURVIVAL AND TOXICITY DATA

C Thomas , Z kassam , N Shah
Mount Vernon Cancer Centre, London, United Kingdom

INTRODUCTION: Gefitinib is an oral selective epidermal growth factor receptor tyrosine kinase inhibitor, which has shown encouraging responses in patients with advanced NSCLC. We have retrospectively analysed the response, survival and toxicity of 33 patients on Gefitinib 250mg daily with progressive NSCLC, treated in the expanded access programme from July 2002 to date.
RESULTS:Median age of 60.6 years (38-78), M:F of 1.1:1, performance status 0/1/2/3: 10/12/9/2.Stage IIIA/IIIB/IV 6/6/21. Tumour response at 2 month using the RECIST criteria showed partial response of 18.8%, stable disease 31.2% and progressive disease in 50.0%. After a mean follow up of 4.8 months (0.5- 12.5 months), 21 patients have died with a mean survival of 4.2 months (0.5- 10 months). Overall symptom improvement was seen in 36%.Using the Common Toxicity Criteria:

| | Grade 1 | Grade 2 | Grade 3 | Grade 4 |
|---|---|---|---|---|
| Diarrhoea | 6 (18%) | 0 | 1 (3%) | 0 |
| Rash | 5 (15 %) | 3 (9%) | 0 | 0 |
| Oral mucositis | 0 | 1 (3%) | 0 | 0 |
| Periorbital oedema | 0 | 1 (3%) | 0 | 0 |
| Anorexia | 1 (3%) | 0 | 0 | 0 |

No nausea, pneumonitis, hepatitis, keratitis were reported. There were no treatment related deaths.
CONCLUSION: Gefitinib monotherapy is well tolerated with encouraging early activity and is a reasonable choice of therapy in pre-treated patients with progressive advanced NSCLC. Detailed toxicity and survival data will be presented at the meeting.

### P22

### AUDIT OF GEFITINIB (IRESSA) IN THE MANAGEMENT OF NON SMALL CELL LUNG CARCINOMA: A SINGLE INSTITUTES EXPERIENCE

DA Power , M Beresford , C Lewanski
Charing Cross Hospital, London, United Kingdom

Gefitinib (IRESSA) is a novel tyrosine kinase inhibitor available on an expanded access programme as oral monotherapy (250mg per day). Early studies (IDEAL1&2) had treatment response rates of 11-18% and ≈40% of patients reported improvement in symptoms.
Aim: to review the use of Gefitinib at Charing Cross Hospital from July 2002 to date.
Results: 31 patients were treated with Gefitinib. Male: female ratio 21: 10. Average age at diagnosis 69 years (range 46 – 79). Stage at time of commencing Gefitinib: 23% stage IIIB (T4 disease), 77% stage IV. Prior treatments: radical radiotherapy 22%; surgery 21%; chemotherapy 70%. Gefitinib was first line treatment in 30%, second line in 43% and 3$^{rd}$ line in 27%. Symptomatic improvement (exercise tolerance and performance score) in 54%. Radiological partial response in 12%; progressive disease in 69%. 1 (3%) complete response (female patient, adenocarcinoma). Median survival from start of treatment = 3 months (range 1 - 12). 6 patients died < 6 weeks from starting treatment, without symptom improvement. Females had improved survival compared to males - median survival: 26 versus 14 months (p=0.004 log rank). No effect of histology on survival. Toxicity: Skin rash (acne, palmer-plantar syndrome) in 67%. Liver enzyme dysfunction in 17%. Diarrhoea; Grade 1 in 36% patients, grade 2 in 4% (1 patient). No pneumonitits. 3 patients (1%) stopped treatment due to toxicity (2 skin toxicity; 2 hepatic toxicity).
Summary: Gefitinib is a well tolerated oral treatment. Efficacy & toxicity profiles appear similar to previous studies. Survival of female patients is better. Timing & use of Gefitinib in this poor prognosis disease requires further investigation. Further survival data will be presented.

### P22:1

### VALIDATING THE PROGNOSTIC VALUE OF MARKER GENES DERIVED FROM A NON-SMALL CELL LUNG CANCER MICROARRAY STUDY

FH Blackhall , DA Wigle , I Jurisica , M Pintilie , FA Shepherd , MS Tsao
United Kingdom, Ontario Cancer Institute, Princess Margaret Hospital and University of Toronto, Toronto

We previously reported that our cDNA microarray analysis of primary non-small cell lung carcinoma (NSCLC) could predict for patients at increased risk of cancer recurrence. From the result of this analysis, we selected 11 genes that were potential candidate prognostic marker genes and used the realtime reverse transcription polymerase chain reaction (RT-PCR) to investigate their expression in the same set of 38 NSCLC cases used for the microarray study.
Cluster analysis of the realtime RT-PCR data for 11 genes separated these patients into two groups with significantly different disease-free survivals (log rank test, p<0.017). However, in a validation series of a further 92 NSCLC cases, cluster analysis failed to confirm the prognostic significance of the realtime RT-PCR results for these 11 genes. In univariate analysis, hypoxia inducible factor 1 alpha, Rho-GDP dissociation inhibitor α and citron/ rho-interacting serine-threonine kinase 21 were significant prognostic factors for disease-free survival in the entire cohort of 130 NSCLC patients, but none were significant in multivariate analysis.
The results demonstrate that the prognostic significance of microarray results can be partially validated using realtime RT-PCR, but secondary validation using larger and independent series of tumours is necessary to identify true prognostic marker genes.

### P23

### AN ASSESSMENT OF THE MODIFICATION OF DIET IN RENAL DISEASE (MDRD) EQUATION FOR ESTIMATING GFR IN CANCER PATIENTS

E-L Bertram [1], LR Campbell [1], CG Fraser [2], P James [3], HP Stevenson [2], EM Rankin [1], MS Highley [1]
[1] Department of Cancer Medicine, Ninewells Hospital, Dundee, United Kingdom, [2] Department of Biochemical Medicine, Ninewells Hospital, Dundee, United Kingdom, [3] Medicines Monitoring Unit, Ninewells Hospital, Dundee, United Kingdom

$^{51}$Cr-EDTA clearance is the gold standard assessment of GFR. However this method is not always available, and a 24 hour creatinine clearance (CC) estimation is often used. The MDRD equation might be useful to estimate GFR in fields other than renal medicine. We have compared CC with the estimated GFR obtained from the MDRD[1] equation and also the Cockcroft and Gault[2] (CG) and Wright[3] (W) formulae, in a retrospective analysis of cancer patients receiving MIC for NSCLC, or ECF for gastric or oesophageal adenocarcinoma. These formulae require only serum and demographic data.
**Methods**: 162 patients (60 females and 102 males) were studied. 126 received MIC and 36 ECF. The median age of all patients was 62 years (range 29-83). For both MIC and ECF, the median number of cycles received was 4 (range 1-6). CC during these treatments was compared to estimates of GFR obtained from MDRD, CG and W, derived from data obtained on the same day as the CC. Creatinine was measured using a kinetic Jaffe method; creatinine kinase measurements were unavailable. There were 105 observations with complete data for CG, and 292 for MDRD and W. A linear regression model was fitted for each of the three formulae.
**Results:** W gave the closest estimates, accounting for 24% of total variance (CG and MDRD accounted for 18% and 5% respectively). There were no significant differences according to whether MIC or ECF was used.
**Conclusion**: The MDRD equation, derived from a patient population with renal impairment, is less predictive of CC in cancer patients than the W formula, derived from a cancer patient population.

1 Levey AS, et al. (1999) *Ann Intern Med* **130**: 461.
2 Cockcroft DW and Gault MH. (1976) *Nephron* **16**: 31.
3 Wright JG, et al. (2001) *Br J Cancer* **84**: 452.

© 2004 Cancer Research UK

### P24

### ARE WE PREVENTING THE PREVENTABLE – VENOUS THROMBOEMBOLISM PROPHYLAXIS IN CANCER

R Jones , S Oliveros , R Deady , RCF Leonard
Cancer Institute, Singleton Hospital, Swansea, United Kingdom

Venous Thromboembolism (VTE) is common in cancer patients and often a cause of death. Post-mortem studies demonstrated rates of VTE as high as 50% in cancer patients. The risk of VTE complications in malignancy is increased by surgery, chemotherapy (including adjuvant) and hormone treatment.

In 2003 we undertook a retrospective audit of 69 consecutive patients admitted to our cancer ward. The purpose was to classify patients according to their VTE risk as defined by the Second Thrombosis Risk Factors Consensus Group risk assessment model into moderate, high and very high risk. [1] The main risk factors in our patients were malignancy, immobility and presence of central venous catheters. Using the risk assessment model 6 of our patients were moderate risk, 37 high risk and 26 patients very high risk. All subsequent events during that admission occurred in the very high risk group with 1 DVT, 1 PE and 2 sudden deaths. None of these patients had received prophylaxis. Only 8 patients (12%) were receiving any type of VTE prophylaxis and only 4 of these were in the very high risk group.

This study identified the need to assess the VTE risk in all our patients and to consider adopting a protocol for prophylaxis with LMWH in the very high risk group and consider TEDS, as less interventional, for the high risk group. This point was emphasised by a recent study of 106 British oncologists, which showed that a quarter did not recognise the thrombogenic effects of cancer treatments and that VTE prophylaxis is not commonly used in patients undergoing treatment for cancer. [2]

1 THRIFT Consensus Group: 1998 Phlebology; 13: 87 – 89
2 Kirwan et al: 2003 BMJ; 327: 597 - 598

### P25

### PHYSICAL ACTIVITY IN CANCER CACHEXIA: DO WE KNOW HOW ACTIVE OUR PATIENTS ARE?

M Dahele [1], L Wall [1], T Preston [2], K Fearon [1]
[1] United Kingdom, University of Edinburgh, Edinburgh, [2] United Kingdom, Scottish Universities Environmental Research Center, East Kilbride

INTRODUCTION: 60-80% of patients with advanced upper gastrointestinal (UGI) cancer are cachectic, increasing morbidity and mortality. Therapy should improve function. It is unclear how active cachectic cancer patients are and what determines their level of activity. It is not known how Performance Status (PS) relates to objective activity, or how best to measure activity. Physical activity is a novel integrative end-point providing objective patient data. This study pilots the use of a commercial activity meter (activPAL professional, PAL Technologies Ltd, Glasgow). The meter is worn mid-thigh and provides detailed activity records including estimated energy expenditure (EEE).
AIMS: To determine if the activPAL meter is a practical tool for assessing activity. To characterise activity in advanced cancer patients.
METHODS: UGI cancer patients with >5% weight loss have their WHO PS recorded and then wear the activPAL meter for 1 week.
RESULTS: Preliminary results from 5 patients. 1 additional patient excluded due to technical failure. All 5 patients had severe cachexia.

| AGE | SEX | WL(%) | WHO | STEP(hrs) | UP(%/16hrs) | EPAL | PPAL |
|---|---|---|---|---|---|---|---|
| 55* | M | 39 | 2 | 7.3 | 18 | 1.1 | 1.5 |
| 59* | M | 19 | 2 | 14 | 36 | 1.2 | 1.5 |
| 62 | M | 19 | 2 | 5.4 | 10 | 1.1 | 1.5 |
| 46* | M | 26 | 1 | 7.8 | 32 | 1.1 | 1.5 |
| 69 | F | 44 | 2 | 5.8 | 17 | 1.1 | 1.5 |

* Receiving chemotherapy, WL Body Weight Loss, STEP Total hours spent Stepping/wk, UP Extrapolated Percentage time spent Standing or Stepping/16hr 'day', EPAL Estimated Physical Activity Level derived from EEE (MET), PPAL Predicted PAL (MET)
SIGNIFICANCE: WHO PS does not reflect patient activity. Stepping time, time UP and EPAL demonstrate the extent of patient inactivity. The meter is well tolerated. The study is ongoing. Additional work will assess activity in a healthy group and validate the EEE function.

### P26

### AUDIT OF WEIGHT LOSS AND NUTRITIONAL SUPPORT DURING RADICAL RADIOTHERAPY OF HEAD AND NECK MALIGNANCIES

SJ Brock , C Baughan , V Hall , J Noyce
Wessex Radiotherapy Centre, Southampton, United Kingdom

**Introduction**. Patients with head and neck malignancy are frequently malnourished at presentation. nutritional status may be further compromised by treatment. the aim of this audit was to assess the weight loss of patients undergoing radical radiotherapy and to introduce guidelines for weight loss management.
**Methods**. audit of weight loss in all patients receiving radiotherapy for head and neck malignancy from february to 0ctober 2001. initial and weekly weights were recorded plus interventions (nil, dietician referral with no other intervention, dietician followed by ng or peg) during radiotherapy. guidelines for weight loss management introduced with the aim of less than 10% of patients losing more than 5% of their initial weight. audit cycle completed between October 2002 and June 2003.
**Results.** 54 patients were identified in the initial audit. the average weight change as a percentage of initial weight was -3.8%(+1.2 to -13.3). 35% of patients lost more than 5% of their initial weight including 56% of patients who saw the dietician without further intervention. on repeat of the audit, 60 patients were identified. the average weight change as a percentage of initial weight was -1.14% (+4.8 to -8.2%). 6.6 % of patients (4 patients) lost more than 5% of their initial weight; all were chemoradiotherapy patients who refused ng tube insertion. intervention rates and percentage weight change according to intervention for the 2 audit spells are shown below.
**Conclusions**. The introduction of guidelines resulted in earlier more effective management of weight loss without increasing the number of interventions.

| Intervention | Rate 2001 | Rate 2003 | Weight change (% Of initial Weight) 2001 | Weight change (% Of initial Weight) 2003 |
|---|---|---|---|---|
| Nil | 50% | 48% | -3.6 (+3.9 to -10.3) | +0.5 (+2.6 to -1.8) |
| Dietician only | 30% | 37% | -5.9 (+3.0 to -3.3) | -2.47 (+0.4 to -8.2) |
| NG / PEG | 20% | 15% | -6.3 (+1.5 to -13.3) | -1.7 (+3.4 to - 4.9) |

### P27

### OPEN LABEL PHASE II STUDY INDUCTION CHEMOTHERAPY USING CISPLATIN OR CARBOPLATIN AND VINORELBINE FOLLOWED BY CONCOMITANT CHEMO-IRRADIATION FOR UNRESECTABLE NON-METASTATIC NON-SMALL CELL LUNG CANCER

S H Bullard [1], G Thomas [2], S Hill [3], L Toy [5], M Collinson [4], P Hajiannakis [1]
[1] Derriford Hospital, Plymouth, United Kingdom, [2] Leicester Royal Infirmary, Leicester, United Kingdom, [3] Pierre Fabre Onocology, Winchester, United Kingdom, [4] Royal Cornwall Hospital, Truro, United Kingdom, [5] Royal Devon and Exeter Hospital, Exeter, United Kingdom

Introduction
A multi-centre study to assess feasibility and tolerability of induction chemotherapy followed by concomitant chemo-irradiation in the treatment of inoperable, but non-metastatic non-small cell lung cancer (NSCLC).
Methods
A minimum of 40 patients (20 on each arm) will be treated under this protocol. They will receive 4 cycles of either Cisplatin and Vinorelbine or Carboplatin and Vinorelbine (platinum agent is dependent on individual patient / physician). Radiotherapy commences on day 1 of cycle 3, to a dose of 66Gy over 6.5 weeks. During the radiotherapy, the chemotherapy agents are reduced by 20 – 40%. Toxicity is assessed throughout the treatment, with treatment delays and dose reductions built into the protocol.
Results
To date 24 patients have been entered into this study; 8 have been, or are being, treated in the Carboplatin and Vinorelbine arm; the remaining 16 with Cisplatin and Vinorelbine. 2 patients have been assessed as ineligible. 9 patients are currently on treatment. In the 13 patients who have completed treatment to date, the best response has been 2 CR, 8 PR, 3SD, 0 PD. 2 patients have proceeded to surgery (one had pathCR). As anticipated, chemotherapy and radiotherapy toxicities have been recorded, but have been manageable. There was no severe pneumonitis. During the post treatment period, to date, a median follow up of 10 (4-17) months has been recorded. During this time, three patients have progressed, one of who has since died of PD. Updated results will be presented at the meeting.
Conclusion
The results to date are encouraging and we continue with active recruitment.

© 2004 Cancer Research UK

### P29

### IDENTIFICATION OF A FAMILIAL PANCREATIC CANCER GENE BY FOCUSING HAPLOTYPE ANALYSIS USING LOH IN SPORADIC CANCER

J Leslie [1], C McFaul [1], J Earl [1], B Azadeh [1], W Prime [1], D Bartsch [2], T Harada [3], N Lemoine [3], R Sibson [4], J Neoptolemos [1], W Greenhalf [1]
[1] The University of Liverpool, Liverpool, United Kingdom, [2] Philipps-University, Marburg, Germany, [3] Hammersmith Hospital, London, United Kingdom, [4] Clatterbridge Cancer Research Trust, Bebington, United Kingdom

Pancreatic Ductal Adenocarcinoma (PDAC) is a highly invasive cancer with poor prognosis accounting for 40,000 deaths per year in Europe. The genetic basis for the particularly aggressive nature of this cancer is not known. Our aim is to identify tumour suppressor genes that are inactivated in pancreatic cancer and so are targets for therapy and diagnosis. We are looking for a gene that is mutated in the germline of patients with a familial predisposition for pancreatic cancer (establishing the genes specificity) and which is frequently mutated in sporadic pancreatic cancers. As a marker we are looking for regions of loss of heterozygosity (LOH).
The European Registry of Hereditary Pancreatitis and Familial Pancreatic Cancer (EUROPAC) has identified over 80 families with an autosomal dominant genetic predisposition to pancreatic cancer (FPC). No gene has been identified in these families, but by analogy with other cancer syndromes it is most likely to be a tumour suppressor and is likely to be affected by somatic mutations in sporadic cases of pancreatic cancer. So we expect to see loss of the non-mutated allele in the sporadic cases (LOH).
EUROPAC in collaboration with the German Familial Pancreatic Cancer registry (FaPaCa) will carry out haplotyping to identify chromosomal regions associated with the disease. One such region has already been identified as the 4q32-34 region, which is associated with pancreatic cancer in a single, large American family. We have carried out haplotype analysis of this region and identified 7 families where the disease gene cannot be associated with 4q32-34. By identifying regions of LOH in sporadic cases we will determine the next region to be haplotyped.
One hundred and eleven H&E stained slides from archived paraffin-embedded tissue blocks were pathologically assessed for PDAC. Sections of 7-10 micron in thickness were cut from each block and needle or laser capture microdissected to isolate the cancerous tissue from the non- cancerous areas. DNA was extracted and quantified using Real Time PCR Light Cycler™ (Roche) and UV spectrophotometry.
Comparative hybridisation using whole genome BAC microarrays from the Sanger centre is being used to identify possible regions with LOH.

### P30

### P53 MUTATIONS IN PATIENTS WITH PANCREATIC CANCER AND CHRONIC PANCREATITIS

L Yan [1], C McFaul [1], N Malats [2], J Threadgold [1], N Howes [1], J Leslie [1], T Wong [1], M Lombard [1], H Smart [1], I Gilmore [1], J Evans [1], P Real [2], J Neoptolemos [1], W Greenhalf [1]
[1] The University of Liverpool, Liverpool, United Kingdom, [2] IMIM, Barcelona, Spain

We have developed a technique for detection of p53 mutations in pancreatic juice. with. We aim to show that p53 mutations in pancreatic juice correspond to p53 mutations in tumour tissue and that these are specific for cancer. We also compared p53 mutation status with the mutation status of K-Ras and methylation of the promoter of the tumour suppressor p16.
Mutant p53 was identified in the juice of 23 (45%) of 51 patients with pancreatic ductal adenocarcinoma (PDAC). 2 (4%) of 49 chronic pancreatitis patients. 51 patients had billiary tract stones none of these patients had p53 mutations. There was no correlation in PDAC between p53 and K-Ras mutations (p=0.25, N=48 Fisher's exact). Matched tissue was available in 23 PDAC cases. In 9 (70%) of 13 cases with p53 mutations in tissue mutations were found in pancreatic juice, in 2 out of 10 cases with no p53 mutation in tissue a p53 mutation was identified in juice. The two chronic pancreatitis patients with p53 mutations were aged 19 and 43 when the samples were taken. The 43 year old patient has since died, this patient also had mutant Ras. The 19-year-old patient has now been followed up for 5 years with no sign of malignancy. This patient had wild type K-Ras in pancreatic juice and no methylation of the p16 promoter. A tissue sample from this patient was tested for p53 mutation and none was identified. Age of onset of PDAC is lower for patients with a p53 mutation than for patients with wild type p53 (this is significant for the subgroup of patients over 70 years of age, logrank p=0.014, N=10 mutant and 9 wild type). Combining both p53 and K-Ras for the over 70 age group indicates a later age of onset with patients with both wild type K-Ras and wild type p53 (p=0.017, N= 7 both mutant, 5 both wild type). For survival after diagnosis there seems little difference between p53 wild type and mutant patients.
**CONCLUSION**: p53 can be detected in pancreatic juice and this corresponds to p53 in tumour of PDAC patients. However, it can also be detected in patients with chronic pancreatitis although less frequently. p53 mutations seem to be associated with patients who develop PDAC soon after the age of 70 but earlier age of onset before this does not seem to be associated with p53.

© 2004 Cancer Research UK

